# Hybrid Pectin/Polydopamine Hydrogels with Photothermal Properties

**DOI:** 10.1021/acsomega.5c02084

**Published:** 2025-05-22

**Authors:** Sarp Kolgesiz, Nevra Ozcelik, Nazim Ege Erdemir, Hayriye Unal

**Affiliations:** † SUNUM Nanotechnology Research Center, 52991 Sabanci University, Istanbul, 34956, Türkiye; ‡ Faculty of Engineering and Natural Sciences, Sabanci University, Istanbul, 34956, Türkiye

## Abstract

Photothermal hydrogels have emerged as versatile materials for applications in biomedicine, environmental remediation, and soft robotics due to their ability to convert light energy into heat. In this study, we developed hybrid pectin/polydopamine (PDA) hydrogels with intrinsic photothermal properties through a simple, scalable approach. Pectin, a naturally occurring and biodegradable polysaccharide, was functionalized with PDA via dopamine self-polymerization under alkaline conditions, followed by Ca^2+^-induced cross-linking to form a hydrogel network. The structural and chemical interactions between pectin and PDA were analyzed by using FTIR and UV–vis spectroscopy, confirming successful functionalization and formation of a hybrid structure. The mechanical and viscoelastic properties of the hydrogels were investigated, revealing that PDA acts as a secondary cross-linker with pectin due to its ability to form hydrogen bonds and π–π interactions, resulting in a more flexible but mechanically weaker cross-linked network. SEM and swelling ratio analysis demonstrated that PDA incorporation resulted in a denser hydrogel network compared with neat pectin hydrogels while retaining their swelling behavior. DSC analyses supported this trend, indicating microstructural disruption and reduced thermal stability at higher PDA levels. The photothermal performance of the pectin/PDA hydrogels was assessed under 808 nm near-infrared (NIR) laser irradiation, showing a significant temperature elevation proportional to PDA content. Furthermore, light-activated antibacterial tests using Staphylococcus aureus confirmed that NIR-triggered heating effectively reduced bacterial viability, achieving a 3-log reduction in the bacterial count for the highest PDA concentration. These findings demonstrate that pectin has been successfully transformed into a photothermal hydrogel matrix through a simple approach and highlight the potential of pectin/PDA hydrogels as biocompatible, light-responsive materials.

## Graphical abstract

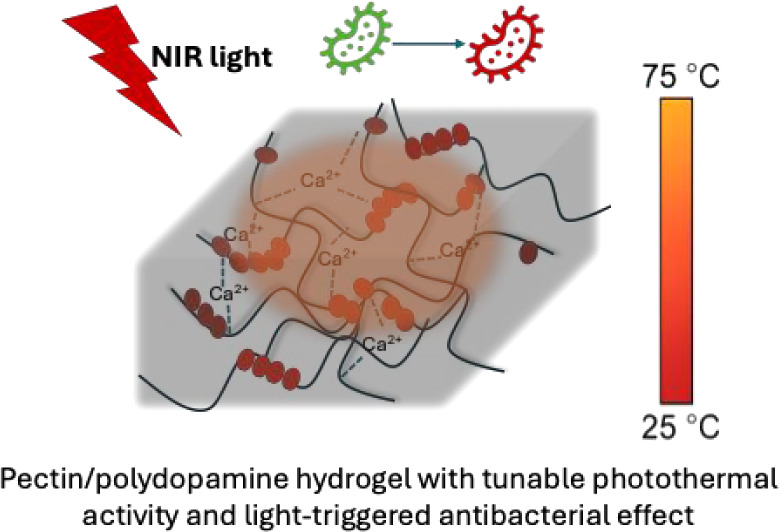

## Introduction

Hydrogels are three-dimensional polymeric networks capable of retaining large amounts of water while maintaining their structural integrity. Due to their high water content, tunable mechanical properties, and biocompatibility, hydrogels have found widespread applications in biomedicine, food, agriculture, environmental remediation, and soft robotics.^1–6^ The unique ability of hydrogels to respond to external stimuli, such as pH, temperature, and ionic strength, makes them particularly useful for controlled drug delivery, tissue engineering, and smart sensing technologies.

A rapidly emerging subclass of hydrogels is photothermal hydrogels, which incorporate photothermal agents that enable the conversion of light energy into heat. These hydrogels exhibit responsiveness to near-infrared (NIR) light, leading to localized temperature changes that can be harnessed for various applications. This thermal responsiveness allows for antibacterial treatments, where localized heating can disrupt bacterial membranes and inhibit microbial growth without harming surrounding healthy tissues.^7,8^ In wound healing and tissue engineering, photothermal hydrogels can promote faster tissue regeneration by enhancing blood circulation and stimulating cellular activities at the wound site.^9–11^ In cancer therapy, photothermal hydrogels can be used for photothermal ablation, where targeted heating destroys cancerous cells while sparing healthy ones, offering a minimally invasive alternative to traditional treatments.^12^ Additionally, these hydrogels are valuable in the development of responsive actuators in soft robotics, where controlled thermal expansion and contraction can drive movement and function in robotic systems.^13,14^ The integration of photothermal functionality into hydrogels significantly enhances their versatility by allowing for remote and on-demand activation, enabling precise spatiotemporal control over their behavior. This characteristic makes photothermal hydrogels highly attractive for a broad spectrum of biomedical and industrial applications, including drug delivery systems, biosensors, environmental remediation technologies, and smart packaging solutions.

Photothermal hydrogels are prepared by incorporating photothermal agents into existing polymer matrices, and they can be classified based on the materials used for photothermal conversion. One major category consists of metallic nanoparticle-based photothermal hydrogels, such as those incorporating gold or silver nanoparticles, which exhibit strong surface plasmon resonance and efficient light-to-heat conversion.^15–17^ Another category is carbon-based photothermal hydrogels, which leverage materials like graphene, carbon nanotubes, or carbon dots for their excellent optical absorption and thermal conductivity.^18–20^ Conducting polymers such as polypyrrole, polyaniline, and polydopamine (PDA) are also being incorporated into hydrogels, resulting in materials favored for their biocompatibility, strong NIR absorption, and ease of functionalization.^21,22^ Conducting polymer-based hydrogels are particularly effective in biomedical applications due to their tunable electrical and thermal properties. MXenes, a class of two-dimensional transition metal carbides, nitrides, or carbonitrides, have recently emerged as promising photothermal agents due to their excellent electrical conductivity, strong NIR absorption, and high photothermal conversion efficiency. MXene-based hydrogels exhibit remarkable mechanical strength, flexibility, and biocompatibility, making them ideal for applications in wearable electronics, biomedical devices, and energy storage systems.^23,24^

While the approach of incorporating photothermal agents into existing polymer matrices in the form of nanoparticles has proven highly effective, it often involves complex synthesis processes, high material costs, and potential environmental concerns due to the use of nonbiodegradable or toxic components. Additionally, this method typically requires careful optimization of the dispersion and stability of nanoparticles within the hydrogel matrix to ensure consistent photothermal performance, which can be challenging and time-consuming. In contrast, there are limited examples of transforming simple, inexpensive, hydrogel-forming polymers into intrinsically photothermal hydrogel-forming matrices, where the base polymer itself or its straightforward modification imparts photothermal properties. This distinction is crucial, as it represents a more sustainable and scalable pathway for developing photothermal hydrogels. Furthermore, intrinsically photothermal hydrogels can offer improved homogeneity and stability compared to nanoparticle-loaded systems, as the photothermal functionality is uniformly distributed throughout the polymer matrix.

In this study, we address this gap by transforming pectin, a readily available and economical biopolymer, into a photothermal hydrogel matrix through the incorporation of PDA. Among the various photothermal agents, PDA has emerged as a particularly attractive candidate due to its strong NIR absorption, adhesive properties, and biocompatibility. PDA mimics the adhesive proteins found in mussels and forms robust coatings on various substrates. Additionally, PDA-based materials can serve as reactive platforms for further functionalization, which expands their application potential. Recent developments in PDA-based hydrogels have demonstrated their efficacy in combining photothermal responsiveness, strong adhesion, UV protection, antioxidant activity, and antibacterial effects, enabling their application in biomedical, environmental, energy, and electronic fields.^25–28^ Here, we present a facile approach to synthesizing pectin/PDA hybrid hydrogels with photothermal properties. Pectin, a naturally occurring polysaccharide, is widely recognized for its biocompatibility, gel-forming ability, and sustainability, making it an ideal candidate for environmentally friendly hydrogel systems. By incorporating PDA into the pectin matrix via a simple reaction of pectin with dopamine under alkaline conditions, we achieve a photothermally responsive hydrogel that can be triggered under NIR irradiation.

## Experimental Section

### Materials

Pectin from citrus peel (galacturonic acid ≥74.0% (CAS number: 9000-69-5)) and calcium chloride (CaCl_2_) were obtained from Sigma-Aldrich Company. Dopamine (3-hydroxytyramine hydrochloride) was purchased from Acros Organics Inc. Ultrapure Tris-base (tris­(hydroxymethyl)­aminomethane) was purchased from MP Biomedicals, LLC. Tryptic soy broth (TSB) and agar powder were procured from Medimark (Italy). Staphylococcus aureus (S. aureus) (ATCC 29213) bacteria were used for the antibacterial activity tests. Milli-Q purified water was used for all synthesis and characterization procedures. All chemicals were utilized as received, without further purification.

### Preparation of Pectin/PDA Solutions

Dopamine at concentrations of 2, 6, and 10 mg/mL was added dropwise to the aqueous 5% pectin solution. After dopamine was added, the pH value of the solutions was adjusted to 8.5 by using ultrapure Tris-base. The reaction time for the self-oxidation polymerization of dopamine was set to 24 h at room temperature. At the end of the reaction time, a dark solution was obtained. The solutions were stored at +4 °C until their next use.

### Preparation of Pectin/PDA Hydrogels

Hydrogels were prepared according to one of the previously reported studies.^29^ For the preparation of native pectin hydrogels, a 12-well plate was utilized as a mold. First, 1 mL of 1 M CaCl_2_ solution was poured into the wells and frozen at −80 °C. The pH of the aqueous 5% pectin solution was adjusted to 8.5 by adding ultrapure Tris-base. Then, 5 mL of the preheated (50 °C) pectin solution was poured into the CaCl_2_-loaded well and placed on a plate shaker overnight to obtain a homogeneous cross-linked hydrogel. After removing the cross-linked pectin hydrogels from the mold, the samples were washed three times with distilled water to remove excess CaCl_2_.

The same procedure was followed to prepare pectin/PDA(2), pectin/PDA(6), and pectin/PDA(10) hydrogels from pectin/PDA solutions prepared with 2, 6, and 10 mg/mL dopamine concentrations, respectively. A 1 mL aliquot of 1 M CaCl_2_ solution was poured into the wells of a 12-well plate and frozen at −80 °C. Then, 5 mL of aqueous pectin/PDA(2), pectin/PDA(6), and pectin/PDA(10) solutions was heated to 50 °C, poured into the CaCl_2_-loaded wells, and placed on a plate shaker overnight to obtain pectin/PDA(2), pectin/PDA(6), and pectin/PDA(10) hydrogels, respectively. After removing the cross-linked pectin hydrogels from the mold, the samples were washed three times with distilled water to remove excess CaCl_2_.

### Characterization of Pectin/PDA Solutions and Pectin/PDA Hydrogels

Fourier transform infrared (FTIR) spectroscopy (Shimadzu IRAffinity-1S), equipped with an ATR system, was utilized to analyze the chemical structure of solutions and hydrogels. All solutions and hydrogels were freeze-dried before chemical structure analysis.

UV–vis spectra were recorded with a spectrophotometer (PG Instruments, T80+) in the range of 200–800 nm after diluting the solutions with distilled water.

The viscoelastic behavior of the hydrogels was analyzed using a rheometer (Physica MCR 301, Anton Paar, Graz, Austria). The storage modulus (*G*’) and loss modulus (*G*″) of the samples were recorded across a strain range from low to high (0.01% to 100%) at a frequency of 1 Hz and room temperature. The tan δ = *G*”/*G*’ value of the samples was also analyzed at each strain rate. The rheometer was equipped with a parallel-plate geometry (diameter: 25 mm; gap: 2.0 mm) to prevent slippage.^30^

The compressive strength of the hydrogels was analyzed with a TA.XTplusC Texture Analyzer (Stable Micro Systems). The force and height calibration of the tool were completed with a 2 kg load cell and automatic confirmation, respectively. To investigate the compressive strength of the hydrogels, the cylinder probe (P/36) was utilized in compression mode.^31,32^ Test parameters were 2 mm/s pretest speed, 0.5 mm/s test speed, 5 mm/s posttest speed, and 5 g trigger force with 60% compression. Thirty mm was adjusted between the baseline and the probe after each test. The maximum value at the compression peak on the stress vs strain (%) graph was reported as the compressive strength of the samples.

The hardness of the samples was investigated by using a TA.XTplusC Texture Analyzer (Stable Micro Systems) equipped with a P/2E probe. After completing force and height calibration of the analyzer, hardness measurements for each sample were conducted with a pretest speed of 2 mm/s, a test speed of 0.5 mm/s, a posttest speed of 5 mm/s, and a trigger force. The maximum value on the hardness graph was recorded as the hardness value of the sample. Three different samples were used to determine the hardness value of the sample.

Differential scanning calorimetry (DSC) measurements were conducted to analyze the thermal transitions of pectin and the pectin/PDA hydrogels. Samples were freeze-dried prior to analysis to ensure the removal of free water. Approximately 5–10 mg of each dried hydrogel sample was accurately weighed and sealed in standard aluminum pans. DSC analysis (Thermal Analysis MDSC TAQ2000) was performed under a nitrogen atmosphere to prevent oxidative degradation. The temperature range was set from 25 to 300 °C with a heating rate of 10 °C/min.

A JEOL JSM-6010 SEM instrument was utilized to investigate the morphology of the hydrogels. All samples were freeze-dried for 24 h and coated with Au–Pd. The SEM images were collected using a secondary electron detector at 5 kV.

Swelling ratios of the hydrogels were evaluated by determining the water content of the hydrogels before and after freeze-drying.^30^ Wet sample weights were recorded after gently removing any surface water. The hydrogels were then freeze-dried for 24 h to measure their dry weights. The experiments were repeated in triplicate. The following formula was utilized to measure the water content of the hydrogels.

1 $$\mathrm{Water content}=\left(W_{o}{-W}_{1}\right){/W}_{1}\times \mathrm{100\%}$$

where *W*_o_ and *W*_1_ represent the weights of wet and dry hydrogels, respectively.

### Photothermal Properties of the Pectin/PDA Hydrogels

The time–temperature profiles of the hydrogels were constructed to examine their photothermal properties. The hydrogel was cut into 1 × 1 × 0.5 cm dimensions and placed in a Teflon holder under an 808 nm laser module (STEMINC, SMM22808E1200) (Doral, FL, USA) at 800 mW/cm^2^ light density. Temperatures were recorded by using a FLIR E6xt thermal camera. Time–temperature profiles were constructed with data obtained from three different spots on each hydrogel; mean and standard error values were reported.

To evaluate the photothermal stability of the pectin/PDA hydrogels, the temperature response of the pectin/PDA(10) hydrogel was measured over three consecutive NIR laser irradiation cycles. A hydrogel sample (1 × 1 × 0.5 cm) was irradiated using an 808 nm NIR laser at a power density of 800 mW/cm^2^ for 5 min per cycle. After each cycle, the sample was allowed to cool to room temperature and was rehydrated in distilled water for 10 min to compensate for water loss due to heating. The surface temperature of the hydrogel was recorded in real time by using an infrared thermal imaging camera.

### Light-Activated Antibacterial Activity of Pectin/PDA Hydrogels

S. aureus (ATCC 29213) cells were cultured in 3 mL of TSB growth medium for 24 h at 37 °C in an incubator with shaking at 200 rpm. The bacteria that had grown were centrifuged, washed twice with sterile Tris buffer (pH 7.5), and resuspended at a concentration of 10^8^ CFU/mL in Tris buffer. 0.5 × 0.5 × 0.2 cm hydrogels were cut and placed in wells of Teflon molds. The hydrogel surface was completely covered with 50 μL of the bacterial suspension. To ensure that the bacteria were not killed by the light alone, the same amount of bacterial suspension was added to an empty well as a control. For each hydrogel sample, two sets of samples were prepared. To examine the NIR light-activated antibacterial properties of the hydrogels, one set was treated with NIR light for 5 min, while the control sample set was kept in the dark for the same amount of time. Bacteria on the hydrogel samples were transferred into 450 μL of Tris buffer by vortexing for 2 min. Bacterial suspensions were serially diluted, plated on TSB agar plates, and incubated at 37 °C for 24 h. Colony counting was performed, and the viability of S. aureus on the hydrogel samples before and after NIR light treatment was reported as log_10_ CFU/mL. Mean and standard error values from 3 different experiments were reported.

## Results and Discussion

The schematic representation of the synthesis of pectin/PDA hydrogels is shown in Figure 1. The first step in the preparation of the photothermal pectin/PDA hydrogels was the self-polymerization of dopamine on pectin chains. Dopamine monomer was mixed with an aqueous pectin solution under alkaline conditions to initiate its oxidative polymerization and the formation of PDA, which was confirmed by the change of the solution color from white to black. Introducing Ca^2+^ ions into the pectin/PDA solution resulted in the formation of a black-colored hydrogel. The physical and chemical interactions leading to the pectin/PDA solutions and pectin/PDA hydrogels were evaluated to elucidate the potential mechanism of the synthesis.

**Figure 1:**
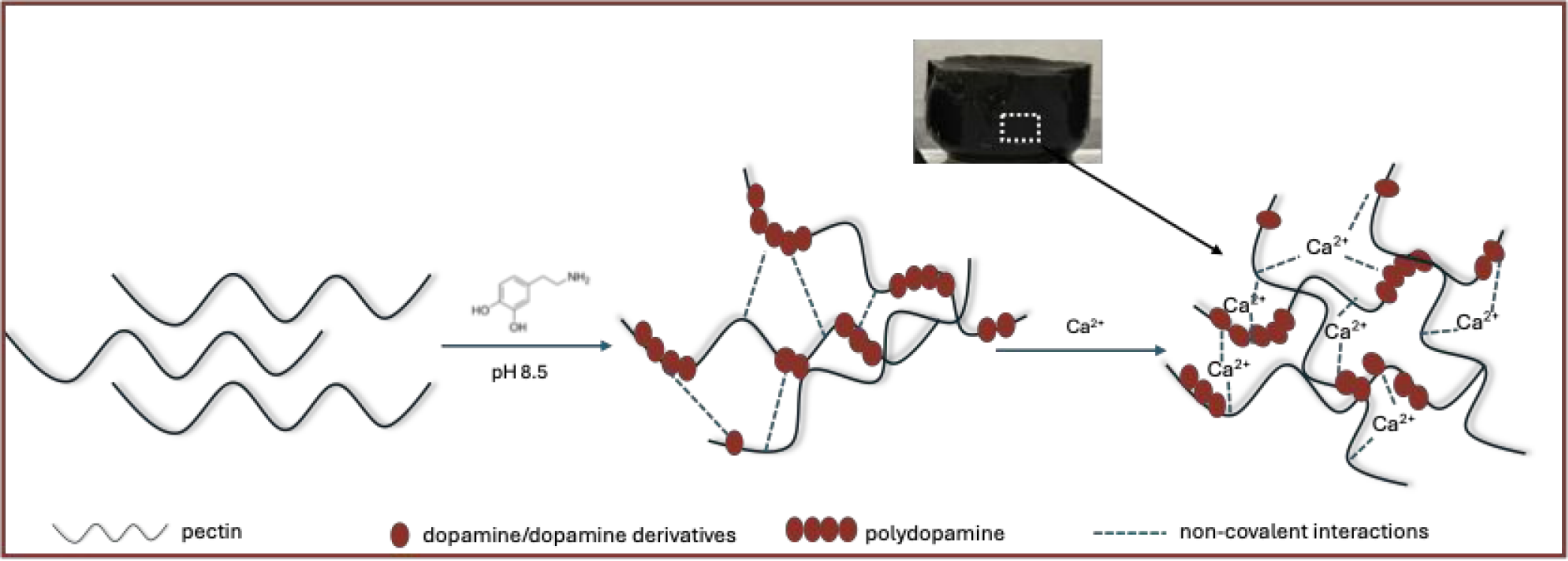
Schematic illustration of the preparation of pectin/PDA hydrogels by alkaline dopamine polymerization in pectin solution followed by Ca^2+^ cross-linking. The inset is a photograph of the pectin/PDA(10) hydrogel.

FTIR spectra of aqueous pectin solutions and pectin/PDA solutions resulting from the reaction of pectin with increasing dopamine concentrations are presented in Figure 2. The broadening and shift of the pectin peak at 3282 cm^–1^ at increasing dopamine concentrations can be attributed to changes in the hydrogen bonding environment, indicating dopamine undergoing oxidation to form quinone and new hydrogen bonding networks being formed between amine, hydroxyl, and quinone groups in the polymer structure. The pectin peak at 2942 cm^–1^ shifts to lower wavenumbers at increasing dopamine concentrations, reflecting changes in the electronic environment of the C–H bonds, potentially due to hydrogen bonding, electrostatic interactions, and covalent or noncovalent bonding with dopamine and PDA, confirming the formation of a pectin/PDA hybrid structure. Furthermore, a new peak at 2853 cm^–1^ emerges likely due to C–H stretching vibrations, possibly amplified by dopamine-pectin interactions at low concentrations. At high dopamine concentrations, dopamine aggregation, polymerization, or binding site saturation may disrupt these interactions, causing the peak to disappear. The peak at 1603 cm^–1^ typically attributed to the asymmetric stretching vibrations of carboxylate groups arising from the presence of carboxy groups in the galacturonic acid units of pectin^33^ presented a shift indicating deprotonation of these groups and their interactions with the dopamine derivatives and PDA. The appearance of a peak at 1517 cm^–1^, associated with the aromatic C=C stretching vibrations, further indicates the incorporation of PDA, whose structure contains aromatic rings from dopamine-derived oligomers. Finally, the disappearance of the peak at 1441 cm^–1^ in the FTIR spectrum of pectin suggests the loss of free −COO^–^ groups, likely due to their involvement in dopamine and PDA interactions. The combination of these spectral changes confirms that pectin is successfully functionalized with PDA and also provides evidence of cross-linking in the pectin/PDA hybrid structure, likely involving hydrogen bonding, resulting in a cross-linked hybrid network.

**Figure 2:**
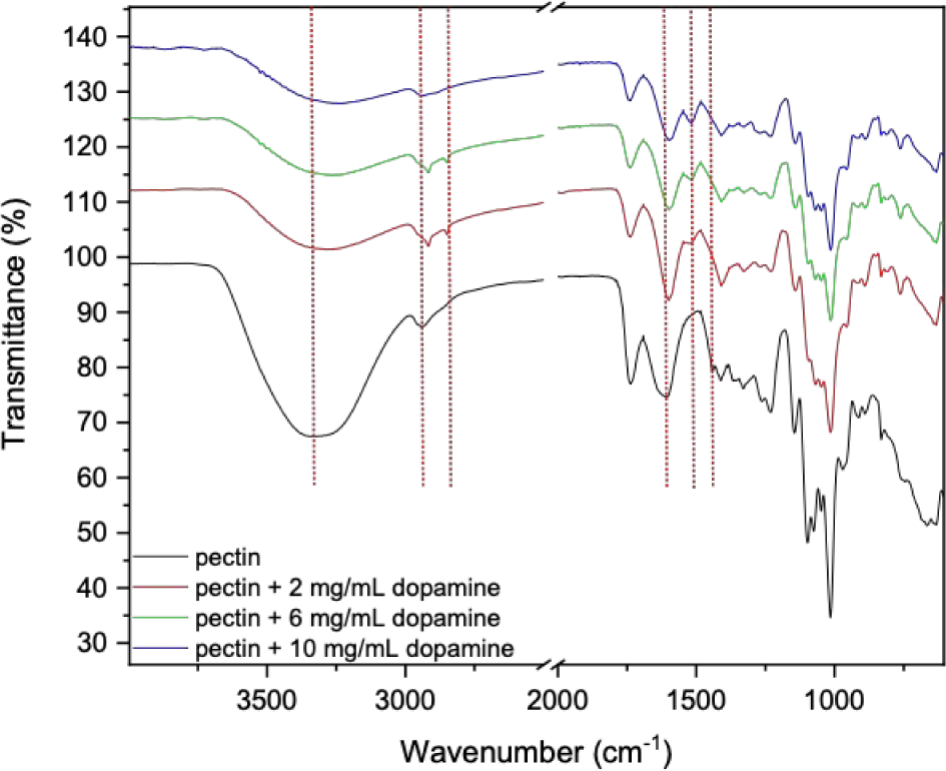
FTIR spectra of pectin and pectin/PDA solutions prepared with different dopamine concentrations (2, 6, and 10 mg/mL). Spectra were recorded to identify chemical interactions between PDA and pectin prior to Ca^2+^ cross-linking.

The UV–vis analysis further confirms the successful incorporation of dopamine into the pectin matrix and the formation of pectin/PDA hybrids (Figure 3). The observed spectral changes, including the increased absorbance at 280 nm and the broadening at 350–450 nm, are indicative of dopamine oxidation, PDA polymerization, and its interaction with pectin. These changes are concentration-dependent, with higher dopamine concentrations leading to greater incorporation and polymerization, resulting in a more stabilized hybrid structure.

**Figure 3:**
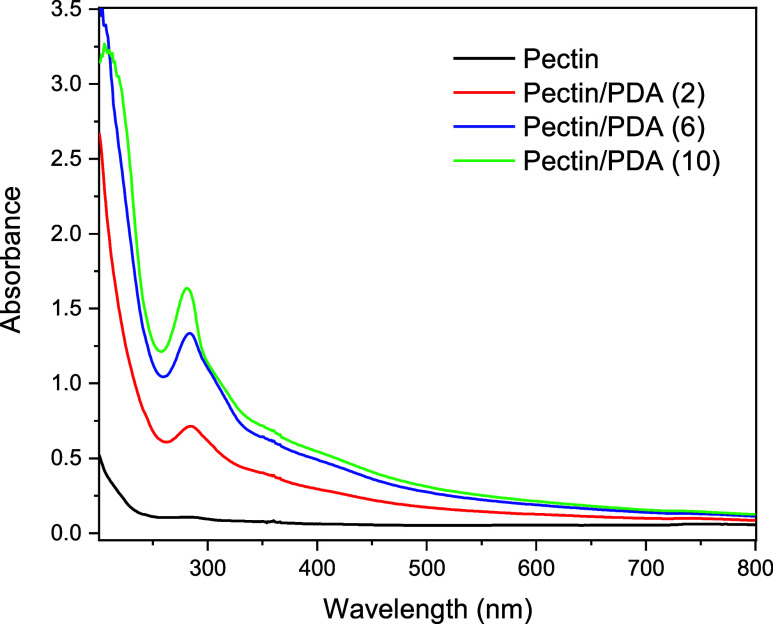
UV–vis absorption spectra of pectin and pectin/PDA solutions. (C, D) Spectra were collected to confirm the oxidative polymerization of dopamine and its integration into the pectin matrix.

The FTIR spectra of pectin/polydopamine hybrids are presented alongside the FTIR spectra of hydrogels formed by reacting these hybrids with Ca^2+^ ions (Figure 4). This comparison highlights the chemical changes that occur during hydrogel formation, facilitated by Ca^2+^ ions. The asymmetric stretching of carboxylate groups at 1603 cm^–1^ shows a slight shift after reaction with Ca^2+^ ions in hybrids prepared with 2 and 6 mg/mL dopamine, which indicates ionic cross-linking between the carboxy groups of pectin and Ca^2+^ ions. In the hybrid with 10 mg/mL dopamine, no significant change is observed, likely due to the limited availability of free carboxy groups as a result of prior interactions with PDA. Similarly, the peak at 1408 cm^–1^, typically associated with the symmetric stretching vibrations of carboxylate groups in pectin, presented a shift only for pectin/PDA hybrids prepared at lower dopamine concentrations indicating that Ca^2+^ ions coordinate with the carboxylate groups and form ionic cross-links. The FTIR spectra demonstrate that cross-linking via Ca^2+^ is more pronounced in pectin/PDA hybrids prepared at lower dopamine concentrations (2 and 6 mg/mL), where more functional groups, such as carboxy and hydroxy groups, remain available for interaction with Ca^2+^ ions. At the highest dopamine concentration (10 mg/mL), the absence of significant spectral changes indicates that the hybrid structure is already stabilized by PDA, leaving fewer reactive sites for Ca^2+^ coordination. These data demonstrate that the facile reaction of pectin with dopamine under alkaline conditions results in a partially cross-linked hybrid pectin/PDA structure, which can be further cross-linked with Ca^2+^ ions into a pectin hydrogel functionalized with PDA.

**Figure 4:**
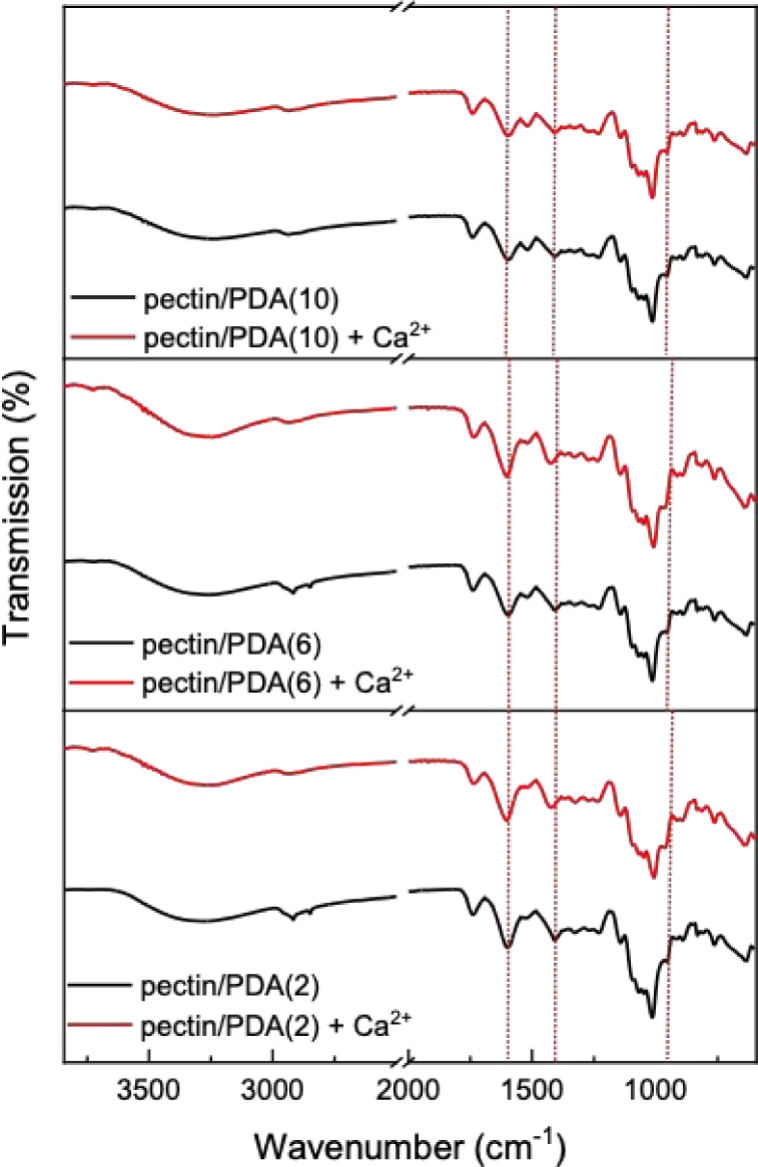
FTIR spectra of dried pectin/PDA solutions prepared at 2, 6, and 10 mg/mL dopamine concentration and pectin/PDA hydrogels prepared by the reaction of pectin/PDA solutions with Ca^2+^ ions.

The viscoelastic properties of the pectin/PDA hydrogels were investigated through dynamic oscillatory rheology, and the results are presented in Figure 5. The storage modulus (*G*’) and loss modulus (*G*’’) were measured to evaluate the stiffness and energy dissipation characteristics of the hydrogel network under oscillatory strain (Figure 5a). *G*’, which represents the elastic behavior of the hydrogel, showed a progressive decrease with increasing dopamine concentration. The decrease in *G*’ at higher dopamine concentrations suggests that excessive polydopamine interferes with the pectin gelation process, leading to a weaker cross-linked network. This may be attributed to steric hindrance or phase separation effects that disrupt the homogeneity of the hydrogel matrix. Similarly, *G*′, which characterizes the viscous nature of the hydrogel, also decreased with increasing dopamine concentration. The reduction in *G*’’ implies that the hydrogel became less capable of dissipating energy under deformation, indicating a decrease in internal friction within the polymer matrix. The shift in *G*’’ to lower values aligns with the observed reduction in *G*’, confirming that higher dopamine content compromises the structural integrity of the hydrogel network. The evolution of tan δ (*G*’’/*G*’) further supports the weakening of the hydrogel with increasing dopamine concentration (Figure 5b). The strain at which tan *δ* equals 1 increases with increasing dopamine concentration, indicating that the material retains its elastic properties over a broader strain range before reaching the crossover point, where viscous behavior dominates. This supports the idea that while the overall stiffness of the hydrogel decreases, the material can sustain elastic deformation for a longer period before transitioning into a flow-dominated regime. This could be due to the formation of a more flexible but weaker cross-linked network, which delays the point at which viscous dissipation overtakes elastic storage.

**Figure 5:**
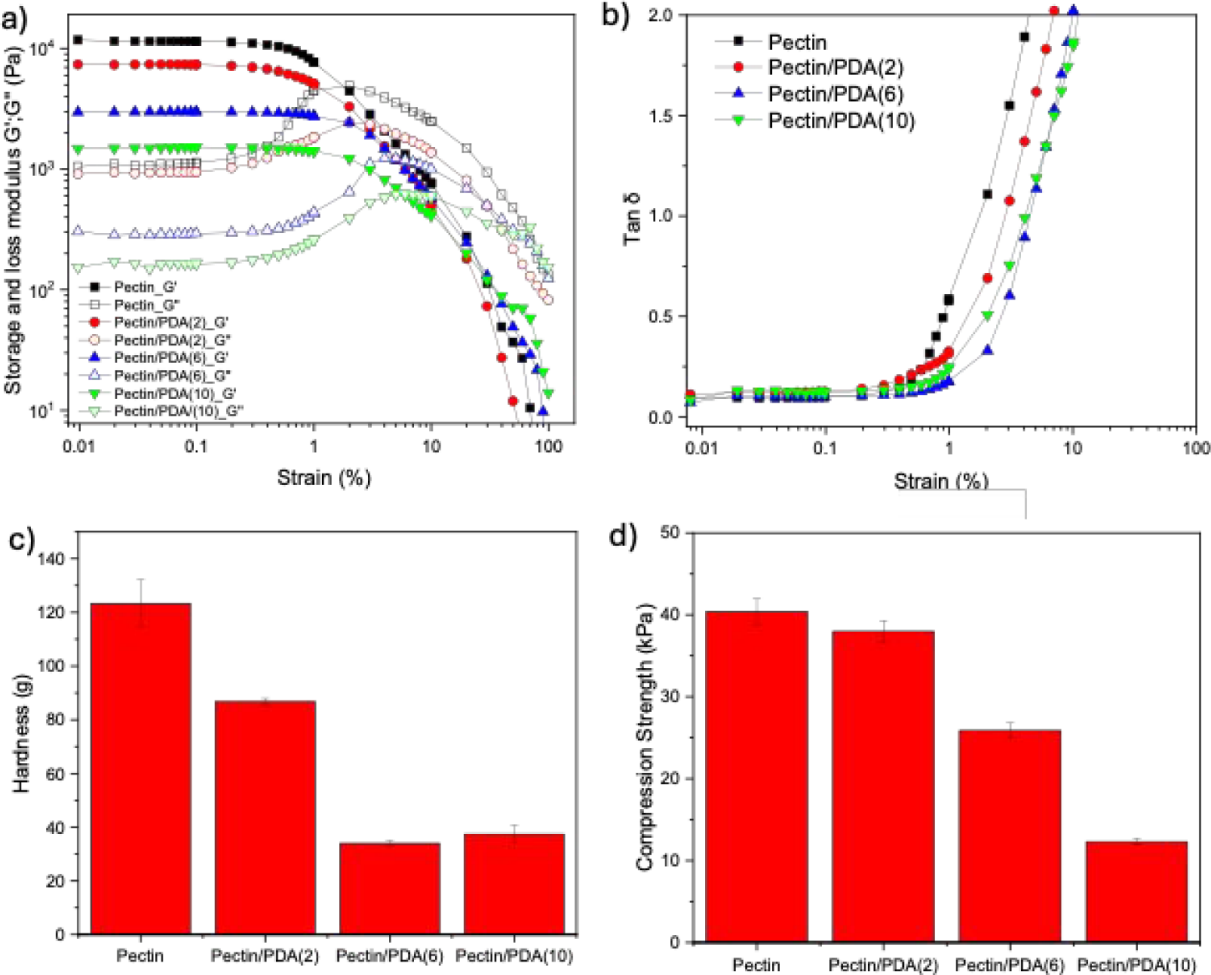
(a, b) Storage modulus (*G*′) and loss modulus (*G*″) of pectin/PDA hydrogels measured as a function of frequency. (c) Hardness and (d) compression strength of pectin and pectin/PDA hydrogels prepared with increasing dopamine concentrations. All hydrogels were cross-linked with Ca^2+^ ions.

The interplay between cross-linking density and mechanical properties explains this behavior. At low dopamine concentrations, polydopamine may introduce additional cross-linking by interacting with pectin through hydrogen bonding and physical entanglement. However, the degree of reinforcement is relatively limited, and the effect on structural integrity is not significantly pronounced compared to calcium-mediated cross-linking. At higher dopamine concentrations, excessive polydopamine may introduce steric hindrance, interfering with the pectin’s gelation process and leading to a less tightly cross-linked, more loosely entangled network. This results in lower stiffness (decreased *G*’ and *G*’’) but greater flexibility (higher strain at tan δ = 1). The hydrogel, therefore, becomes more deformable before reaching its viscoelastic transition, although it ultimately fails under lower stress due to the weaker overall cross-linking.

The hardness (Figure 5c) and compression strength (Figure 5d) values of pectin/PDA hydrogels prepared with increasing dopamine concentrations were consistent with the rheology data, showing that the hardness and compression strength of the hybrid pectin/PDA hydrogels were lower than those of the pectin hydrogel, and the mechanical strength decreased with increasing PDA content. The slight fluctuations, such as pectin/PDA(6) having similar hardness values as pectin/PDA(10), potentially arise from microphase separation or heterogeneous PDA distribution at higher dopamine concentrations. A similar trend indicating decreased mechanical strength with increasing PDA concentration has been observed in hybrid silk fibroin/PDA hydrogels prepared via interactions of silk fibroin with dopamine, where the Young’s modulus values of the hydrogels decreased with increasing dopamine concentrations.^34^ The mechanical analysis further demonstrates that an increased amount of PDA compromises the overall mechanical integrity, which can be attributed to variations in cross-linking density, where excessive polydopamine incorporation reduces effective Ca^2+^-cross-linking and creates a more deformable yet structurally weaker hydrogel network. To address this limitation, alternative reinforcement strategies such as enzymatic cross-linking or dual cross-linking approaches combining ionic and covalent bonding, which have shown promise in maintaining both mechanical strength and responsiveness in other hydrogel systems, can be explored.^35,36^ Although the current study focuses on initial viscoelastic and compression behavior, additional assessments such as self-healing ability, creep-recovery, and stress-relaxation tests could offer further insights into long-term structural resilience and durability—particularly for biomedical applications requiring repeated mechanical loading. (Figure 6)

**Figure 6:**
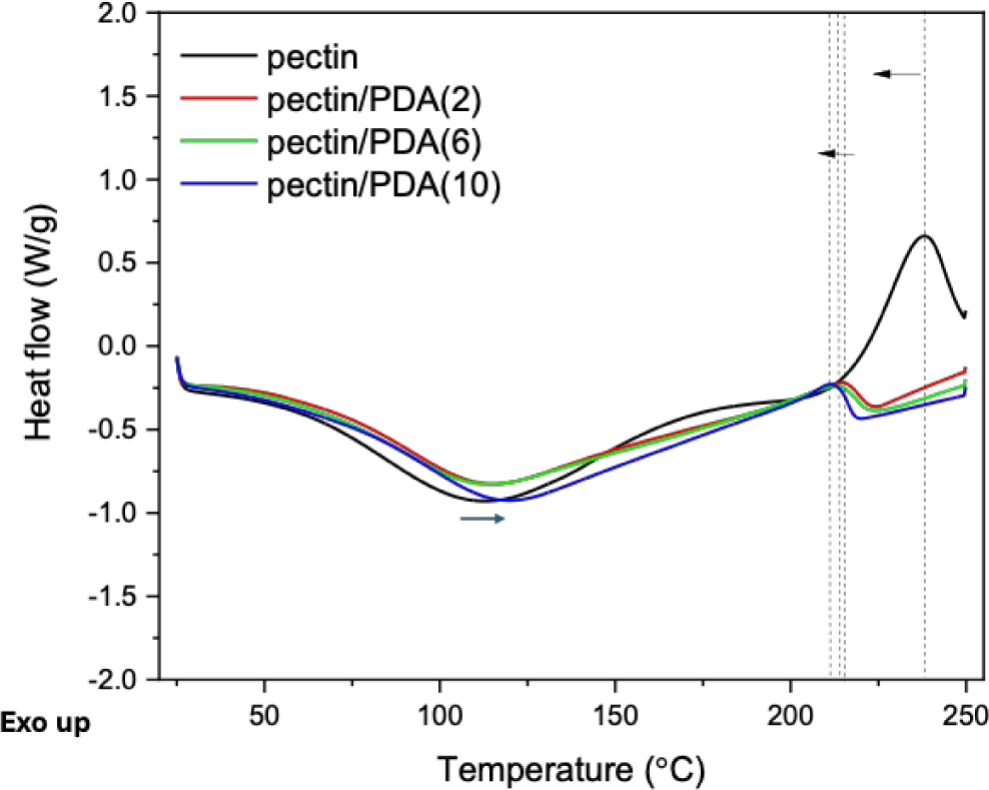
DSC thermograms of Ca^2+^-cross-linked pectin and pectin/PDA hydrogels prepared by the cross-linking of pectin/PDA(2), pectin/PDA(6), and pectin/PDA(10) solutions with Ca^2+^ ions.

The thermal properties of pectin and pectin/PDA hydrogels cross-linked with Ca^2+^ were evaluated by DSC to assess changes in network stability upon PDA incorporation. All samples exhibited an endothermic peak between ∼90–120 °C, attributed to the evaporation of bound and free water within the hydrogel matrix. In PDA-containing hydrogels, this dehydration peak shifted slightly to higher temperatures, suggesting enhanced water retention due to hydrogen-bonding interactions between water molecules and PDA’s catechol or amine groups. The increased thermal resistance to water loss indicates a modified hydration environment in the hybrid network. Additionally, a broad exothermic peak corresponding to the thermal degradation of the pectin backbone was observed between ∼220 and 260 °C. Notably, this degradation peak appeared at lower temperatures and with reduced intensity in the PDA-containing samples, particularly at higher PDA concentrations. This behavior suggests that PDA incorporation disrupts the Ca^2+^-mediated cross-linking and induces a more amorphous, thermally less stable network. The lower enthalpy and earlier onset of degradation may also reflect the altered packing structure and catalytic influence of PDA’s redox-active functional groups. Collectively, these results confirm that PDA significantly influences the thermal behavior of the pectin hydrogel system, not only in terms of the degradation profile but also in water–matrix interactions.

To further elucidate the impact of PDA incorporation on the microstructure of the pectin/PDA hydrogels, SEM analysis was conducted (Figure 7). The larger pore sizes of the pectin hydrogel decreased as the PDA content increased in the hybrid pectin/PDA hydrogels, and the network appeared to be denser. This structural compaction is indicative of stronger interactions between PDA and pectin, likely due to enhanced hydrogen bonding and π–π interactions. For the pectin/PDA (10) (Figure 7d), the structure exhibits the most compact arrangement with minimal visible porosity. However, closer examination reveals fragmented and discontinuous pore walls, suggesting that excessive PDA disrupts the native gelation of pectin rather than reinforcing it. This observation is consistent with the mechanical testing results, which showed a decline in compressive strength at higher PDA concentrations. PDA potentially interferes with pectin’s Ca^2+^ cross-linking, disrupting the polymer matrix, which reduces interchain interactions in pectin, making the hybrid material more fragile. Excess PDA may also lead to phase separation or agglomeration, creating weak points in the material. Thus, PDA acts as a secondary cross-linker with pectin due to its ability to form hydrogen bonds and π–π interactions, which tightens the network and reduces the average pore size. However, at the same time, it competes with Ca^2+^ for interaction sites, reducing the efficiency of cross-linking and leading to a mechanically weaker structure.

**Figure 7:**
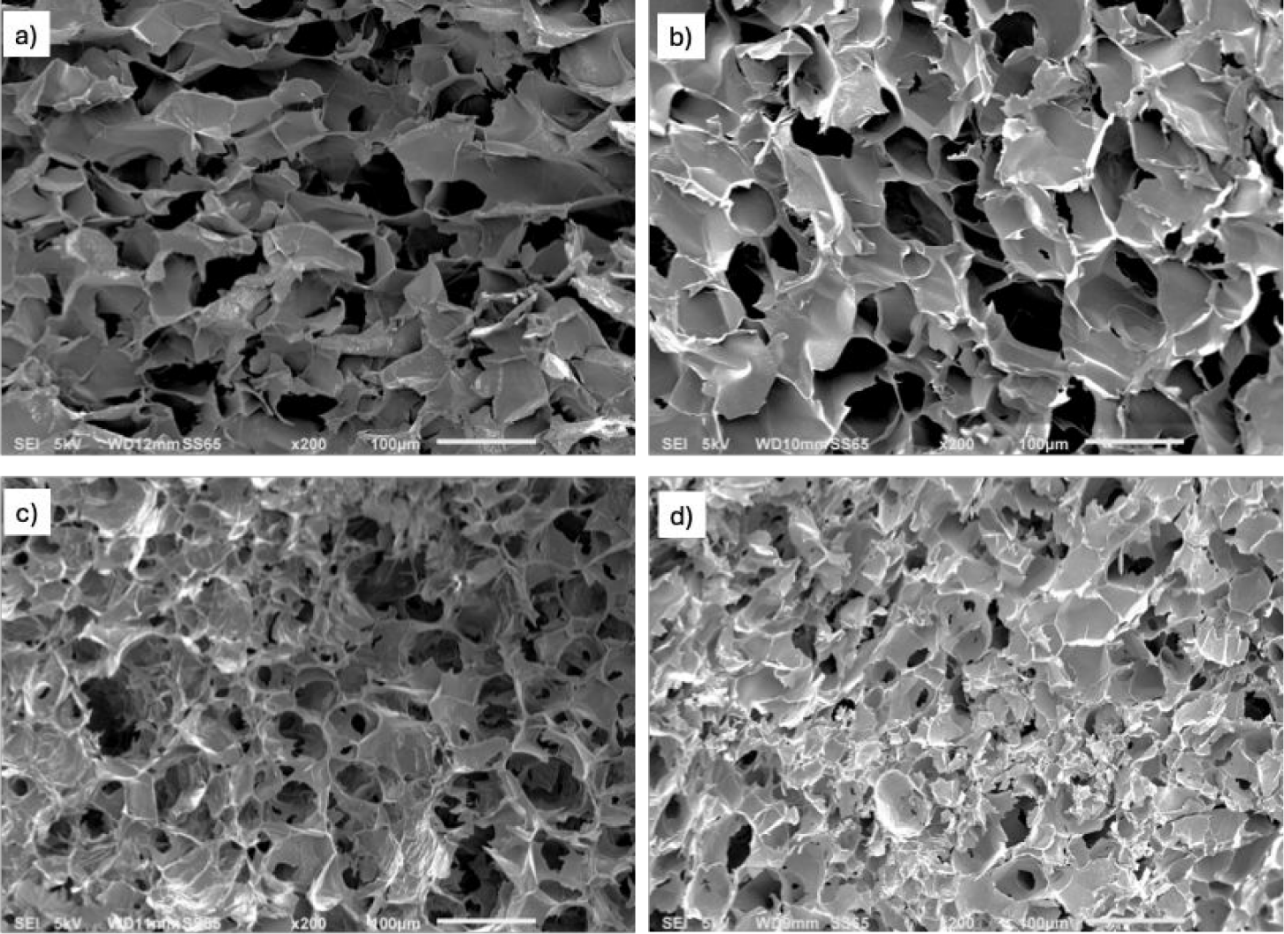
SEM images of (a) pectin, (b) pectin/PDA(2), (c) pectin/PDA(6), and (d) pectin/PDA(10) hydrogels.

The swelling behavior of the pectin/PDA hybrid hydrogels with increasing PDA content is shown in Figure 8. The pristine pectin hydrogel exhibits a relatively high swelling ratio, indicative of its highly porous network and hydrophilic nature, which allows significant water absorption. This is consistent with the large pore size observed in the SEM images, which facilitates water penetration and retention within the hydrogel matrix. The swelling behavior of the hydrogels was influenced by the presence and concentration of PDA. An initial increase in swelling was observed at lower PDA concentrations (e.g., pectin/PDA(2)), which may be attributed to partial disruption of the pectin network and the introduction of additional hydrophilic sites, resulting in increased porosity and water uptake. However, at higher PDA concentrations (pectin/PDA(6) and pectin/PDA(10)), the swelling ratio decreased. This could be attributed to a disrupted network structure, as supported by the fragmented pore walls observed in SEM images and further confirmed by mechanical data, indicating a compromised network integrity rather than uniform reinforcement. The presence of hydrophobic PDA domains and possible aggregation may cause localized physical compaction, limiting water penetration. Similar behavior has been reported in other PDA-containing systems, where higher PDA content led to reduced equilibrium swelling due to hydrophobic interactions and network densification through noncovalent associations.^37^

**Figure 8:**
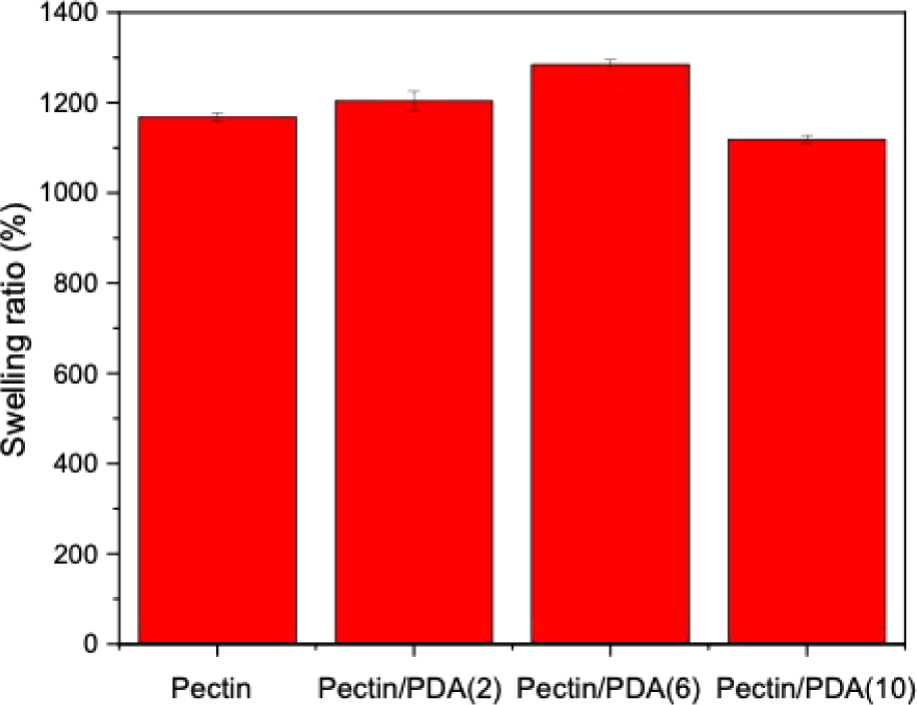
Swelling ratios of pectin, pectin/PDA(2), pectin/PDA(6), and pectin/PDA(10) hydrogels.

An overall assessment of the structure–property relationship suggests that PDA incorporation modulates both the gelation behavior and the structural organization of the pectin hydrogel through noncovalent interactions. At low to moderate concentrations, PDA likely enhances physical cross-linking via π–π stacking and hydrogen bonding with pectin, leading to increased porosity, water uptake, and slight mechanical reinforcement. In contrast, higher PDA content disrupts the network, likely due to steric hindrance, competition with Ca^2+^ for cross-linking sites, and possible phase separation. This is reflected in reduced swelling, compromised mechanical integrity, and irregular microstructures at elevated PDA levels. The shift of the thermal degradation peak to lower temperatures in PDA-rich samples further supports the conclusion that excessive PDA destabilizes the hydrogel matrix. Overall, these results point to a dual role of PDA: as a functional cross-linker at low concentrations and a disruptive agent at higher loadings.

Whether the incorporation of PDA has imparted photothermal properties to pectin as expected was evaluated by constructing time–temperature profiles of pectin/PDA hydrogels under NIR laser irradiation (Figure 9a,b). NIR laser-irradiation-triggered temperature elevations were monitored for pectin and pectin/PDA hydrogels prepared at increasing dopamine concentrations. As shown in Figure 8, the irradiation of the neat pectin hydrogels under the NIR laser did not cause any temperature elevations within 5 min, whereas the pectin/PDA hydrogels were heated significantly under the same conditions due to their photothermal character. The degree of light-triggered temperature increases was observed to be in direct proportion to the PDA content of the pectin/PDA hydrogels. The temperature of the pectin/PDA(10) hydrogel, with the highest content of PDA, reached 75 °C when irradiated with a NIR laser for 5 min. Thus, it has been shown that the photothermal effect of PDA was transferred to pectin and that the degree of light-triggered heating of the pectin/PDA hydrogels can be adjusted by changing the amount of integrated PDA via adjusting the starting dopamine concentrations. It was demonstrated that the pectin/PDA hydrogels can be remotely heated by NIR irradiation and utilized for different applications where remote photothermal heating would be useful.

**Figure 9:**
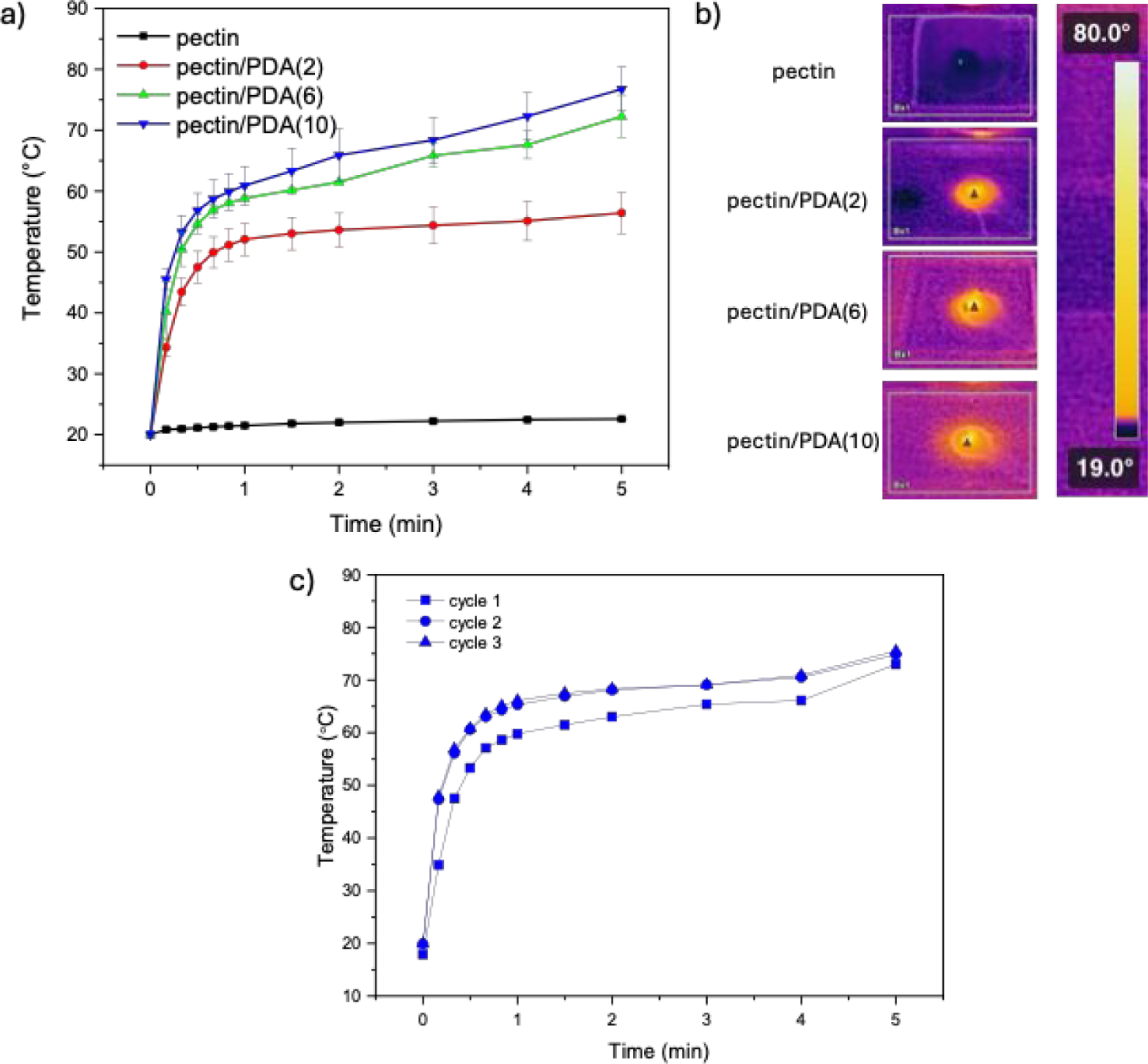
(a) Temperature profiles of pectin and pectin/PDA hydrogels containing 2, 6, and 10 mg/mL dopamine under 808 nm NIR laser irradiation (800 mW/cm^2^) for 5 min, (b) infrared thermal images of the hydrogel surfaces after min of NIR exposure, and (c) photothermal stability of the pectin/PDA(10) hydrogel over three successive NIR irradiation cycles (5 min per cycle) with rehydration between cycles.

To evaluate the reusability and photothermal stability of the pectin/PDA hydrogels, repeated NIR irradiation cycles were performed on the pectin/PDA (10) hydrogel with temperature changes monitored over five successive on/off laser cycles (Figure 9c). After each 5 min irradiation, the hydrogel was allowed to cool to room temperature and rehydrated to compensate for water loss due to heating. The hydrogel consistently reached similar maximum temperatures in each cycle, with only minor variation, indicating excellent photothermal repeatability and structural stability under repeated NIR exposure. This stable temperature profile confirms that the photothermal performance of the PDA-containing hydrogel is retained over multiple cycles, with no significant photobleaching, degradation, or loss of functionality. These results suggest that the pectin/PDA (10) hydrogel can be used for repeated on-demand photothermal activation in biomedical or antimicrobial applications without loss of efficiency.

The utilization of pectin/PDA hydrogels, which present unique light-to-heat conversion properties as materials capable of killing bacteria via light-activated heating, was investigated. The viability of an aqueous suspension of S. aureus dropped on the pectin and pectin/PDA hydrogels was examined before and after NIR irradiation of the hydrogels (Figure 10). Irradiation of the neat pectin hydrogels that did not contain PDA resulted in insignificant antibacterial activity. On the other hand, the number of viable S. aureus dropped on the pectin/PDA hydrogels decreased significantly after 5 min of NIR laser light irradiation, with the pectin/PDA(10) hydrogel containing the highest PDA content resulting in a 3-log reduction in the number of bacteria. The fact that suspensions of S. aureus that were exposed to the same irradiation conditions did not exhibit any bacterial killing confirmed that the bacteria were not directly killed by light. In the absence of NIR irradiation, the pectin/PDA(10) hydrogel exhibited a slight reduction in bacterial viability compared to the control, suggesting that PDA incorporation may confer inherent antibacterial properties. This observation aligns with previous reports indicating that PDA possesses mild antimicrobial effects, potentially due to its surface-active catechol/quinone groups, which can disrupt microbial membranes or interact with bacterial proteins.^38,39^ However, this effect was relatively modest in our system and became pronounced only upon NIR-triggered photothermal activation. These findings confirm that the antibacterial performance of the pectin/PDA hydrogels primarily stems from light-induced heating but may be partially supported by intrinsic PDA activity, particularly at higher concentrations. Consequently, hybrid pectin/PDA hydrogels demonstrated remarkable NIR light-activated antibacterial properties in in vitro testing and exhibit strong potential for use as hydrogels that can be remotely purified of microorganisms via NIR irradiation.

**Figure 10:**
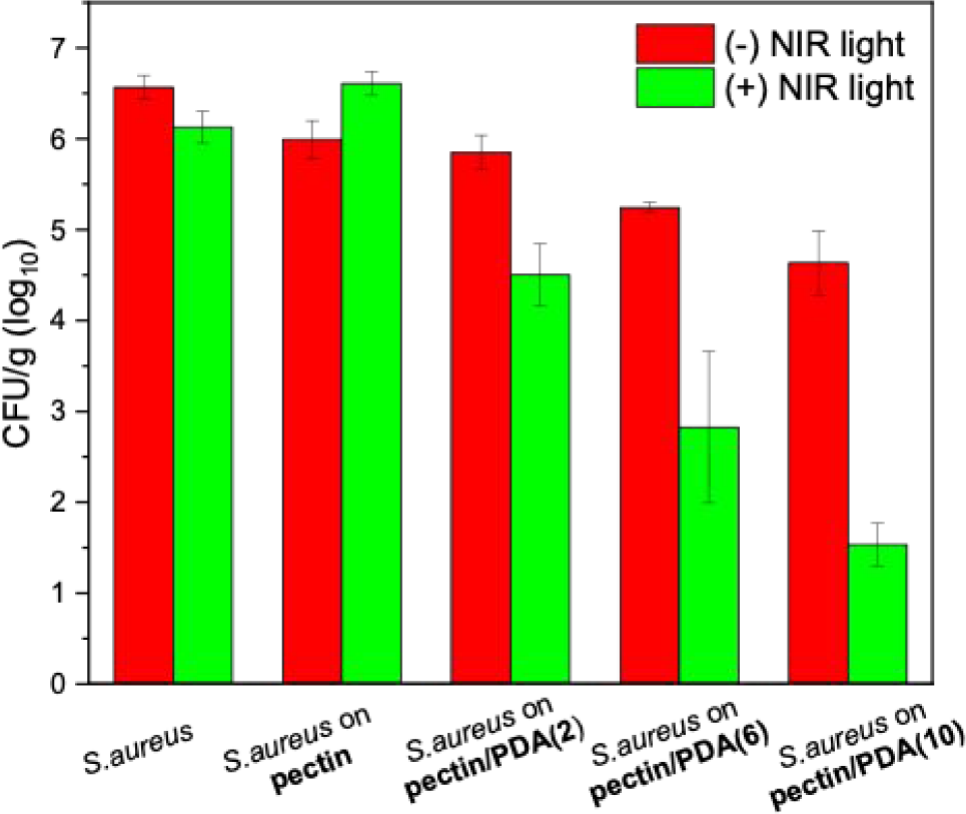
Viability of S. aureus suspension on pectin, pectin/PDA(2), pectin/PDA(6), and pectin/PDA(10) hydrogels before and after 5 min of NIR laser irradiation.

## Conclusions

In this study, we successfully synthesized pectin/PDA hydrogels that exhibit tunable photothermal properties, achieved through a simple and scalable dopamine polymerization approach. The incorporation of PDA within the pectin matrix was confirmed via FTIR and UV–vis spectroscopy, showing strong interactions between the pectin and PDA functional groups. The hybrid hydrogel system demonstrated compromised mechanical strength at higher PDA concentrations, attributed to reduced Ca^2+^ cross-linking efficiency. Rheological and compression tests further confirmed that excessive PDA incorporation led to a weaker but more flexible hydrogel network. The photothermal properties of the pectin/PDA hydrogels were successfully validated, with increasing PDA content enhancing the light-to-heat conversion under NIR irradiation. The highest PDA-loaded hydrogel (pectin/PDA(10)) exhibited a temperature rise of up to 75 °C, demonstrating excellent potential for photoresponsive applications. Moreover, these hydrogels exhibited remarkable light-activated antibacterial properties, as evidenced by a significant S. aureus reduction upon irradiation. These findings highlight the potential of pectin/PDA hydrogels as sustainable, biocompatible, and versatile materials for light-responsive biomedical applications requiring remote heating. The fact that PDA is well-known for its drug-binding capacity through π–π stacking and hydrogen-bonding interactions makes it a promising candidate for dual-function systems. While the current study focuses on establishing the photothermal and antibacterial performance of pectin/PDA hydrogels, incorporating drug-loading capabilities could significantly broaden their application potential, particularly for combined photothermal and chemotherapeutic treatments. Future studies will focus on exploring the feasibility of simultaneous drug delivery and NIR-triggered therapy by using this platform. Investigating degradation kinetics and biocompatibility in physiological environments will also be critical for future biomedical applications.
